# Datasets on how misinformation promotes immune perception of COVID-19 pandemic in Africa

**DOI:** 10.1016/j.dib.2020.106031

**Published:** 2020-07-17

**Authors:** Olalekan Akintande, Olusanya Olubusoye

**Affiliations:** a ACADEMY Mobility Project, Kenyatta University, Kenya & Laboratory for Interdisciplinary Statistical Analysis, Department of Statistics, University of Ibadan, Nigeria; b Laboratory for Interdisciplinary Statistical Analysis, Department of Statistics, University of Ibadan, Nigeria

**Keywords:** Immune perception, COVID-19 or 2019-nCoV, Misinformation, Confirmatory factor analysis, Africa

## Abstract

The dataset investigates the magnitude of the misinformation content influencing scepticisms about the novel COVID-19 pandemic in Africa. The data is collected via an electronic questionnaire method and twenty-one Africa countries randomly participated. Responses were received from all the five regions of Africa. The data is structured to identify some leading misinformation been propagated in the media. For data, in brief, we performed a descriptive analysis of the data and also examine the degree of each selected misinformation contents on the immune perception of respondents using Confirmatory Factor Analysis. Another research can use the dataset to investigate how misinformation and religion misconception promote ignorance about disease or pandemic in Africa or the dataset could serve as supplementary material for further investigation of COVID-19 pandemic in Africa.

Specifications Table

**Table:** 

Subject	Decision Science
Specific subject area	Investigating how misinformation contents about the pandemic promote scepticism.
Type of data	Table Figure Analyzed (misinformation content/items) Raw from the survey (all questions including demographic information)
How data were acquired	Electronic survey shared via social media: Whatsapp and email, available at: Link to Questionnaire, raw data and analyzed data: https://data.mendeley.com/datasets/chrc52k4f5/draft?a=67edb027-ecb5–4278–9cac-97f0ef735900
Data format	Raw from the internet platform used for the collection. We attached the analyzed and filtered as supplementary data.
Parameters for data collection	The descriptive statistics examine the magnitude of misinformation and the statistical significance of each content. The confirmatory factor analysis examines misinformation content contribution to immune perception or scepticisms about the pandemic in Africa.
Description of data collection	The data is collected via an electronic questionnaire. As a dataset limitation, we are unable to cover a very large population (*n* = 563 responses), however, the sample space of the respondents covers all the regions of Africa and we received responses from 21 Africa countries.
Data source location	We survey from the Kenyatta University, Kenya in conjunction with the Laboratory for Interdisciplinary Statistical Analysis (UI-LISA), Department of Statistics, University of Ibadan, Nigeria. We shared the survey via an online platform in adherence with WHO and government directive on social/physical distancing and the survey covers 21 Africa countries (listed under the experimental design, material, and methods section). The raw data is available on the Mendeley repository with the link below Mendeley: https://data.mendeley.com/datasets/chrc52k4f5/draft?a=67edb027-ecb5-4278-9cac-97f0ef735900
Data accessibility	Mendeley: https://data.mendeley.com/datasets/chrc52k4f5/draft?a=67edb027-ecb5-4278-9cac-97f0ef735900 NB: questionnaire for the data is provided in the supplementary file.

**Value of the Data**

• The dataset will enable readers/users to understand how misinformation promotes scepticisms about the pandemic (COVID-19) and towards adherence to preventive measures as well as causes of spread.
• The data method adopted helps identify the most conceived and promoted misinformation about COVID-19 pandemic in Africa [1].
• Since misinformation misleads and could cause much havoc in time of the pandemic, it is important to world health bodies to understand the trend and danger it poses to plan for future occurrence.
• Another research can use the dataset to investigate how misinformation and religion misconception (this was not considered in this dataset but it is available in the raw data) promote ignorance about disease or pandemic in Africa [1,2].
• The dataset could also serve as supplementary material or template for further investigation of COVID-19 pandemic and related issues in Africa [3].

## 1 Data description

The data is collected via an electronic questionnaire. For the data used, we title it as “misinformation” in the repository. The data is an extraction from the raw dataset (named as Perception of COVID-19 disease in the repository. The perception data is the raw response received from the survey) which contains mainly questions (i.e., the ten-misinformation content/items are used) that address the misinformation about the COVID-19 pandemic in Africa. All the questions are coded on a five-point Likert scale. Table 1 presents the procedure or method used to score and classify each misinformation item/content as either promoting immune perception (score = 4) or not promoting immune perception (score = 0). Table 2 presents the descriptive result; the magnitude of misinformation items, which classifies each misinformation content/item as either promoting ignorance or not promoting ignorance.

**Table 1:** Question module.

Participant response per item	Strongly agree	Agree	<= Neutral =>	Disagree	Strongly disagree
*type = "Periodical" Score*	4	4	1	0	0
*Item*	Promotes ignorance about the pandemic		Neither	Does not promotes ignorance about the pandemic	
*Range*	40		10	0

**Table 2:** Magnitude of misinformation scale.

Table 3 presents the statistical significance of each misinformation content. That is, it presents individual strength or magnitude of each misinformation item in promoting ignorance of the COVID-19 pandemic in Africa. Table 4 presents the joint influence (correlation) of the misinformation content/item in promoting ignorance of COVID-19 in Africa.

**Table 3:** Test of significance.

Item/misinformation content	Chi-square	*P*-value
African Blood compositions resist COVID19.	150.819	0.000
Africa Weather and humid system prevent the spread of COVID19?	109.645	0.000
Africans are naturally resilient and resistant to most diseases.	122.792	0.000
Black skin resists COVID19.	271.151	0.000
Older people are most prone to the danger of COVID19, younger people are less prone.	437.405	0.000
Alcohol consumption can prevent/resist/kill COVID19.	241.093	0.000
Drinking hot water can prevent/resist/kill COVID19.	173.332	0.000
Smoking weeds (Cannabis) can prevent/resist/kill COVID19.	388.641	0.000
High-temperature cure COVID19.	80.031	0.000
China made the novel Coronavirus (COVID19) to become the global power	125.147	0.000

**Table 4:** Correlation analysis.

	*Item 1*	*Item 2*	*Item 3*	*Item 4*	*Item 5*	*Item 6*	*Item 7*	*Item 8*	*Item 9*	*Item 10*
Item 1	1									
Item 2	**0.589**	1								
Item 3	0.457	0.475	1							
Item 4	**0.653**	**0.551**	**0.592**	1						
Item 5	0.079	0.138	0.080	0.026	1					
Item 6	0.350	0.348	0.259	0.354	0.028	1				
Item 7	0.330	0.368	0.327	0.331	0.043	0.439	1			
Item 8	0.286	0.243	0.169	0.289	−0.052	**0.591**	0.295	1		
Item 9	0.308	0.389	0.249	0.329	0.0024	0.400	0.486	0.281	1	
Item 10	0.161	0.180	0.218	0.183	−0.043	0.075	0.168	0.047	0.162	1

*Key: Item1***:** African Blood compositions resist COVID19, *Item2 –* Africa Weather and humid system prevent the spread of COVID19, *Item3* – Africans are naturally resilient and resistant to most diseases, *Item4*: Black skin resists COVID19, *Item5***–** Older people are most prone to the danger of COVID19, younger people are less prone, *Item6***:** Alcohol consumption can prevent/resist/kill COVID19, *Item7***–** Drinking hot water can prevent/resist/kill COVID19, *Item8***:** Smoking weeds (Cannabis) can prevent/resist/kill COVID19, *Item9***-** High-temperature cure COVID19, *Item10***-** China made the novel Coronavirus (COVID19) to become the global power.

**Fig. 1:**
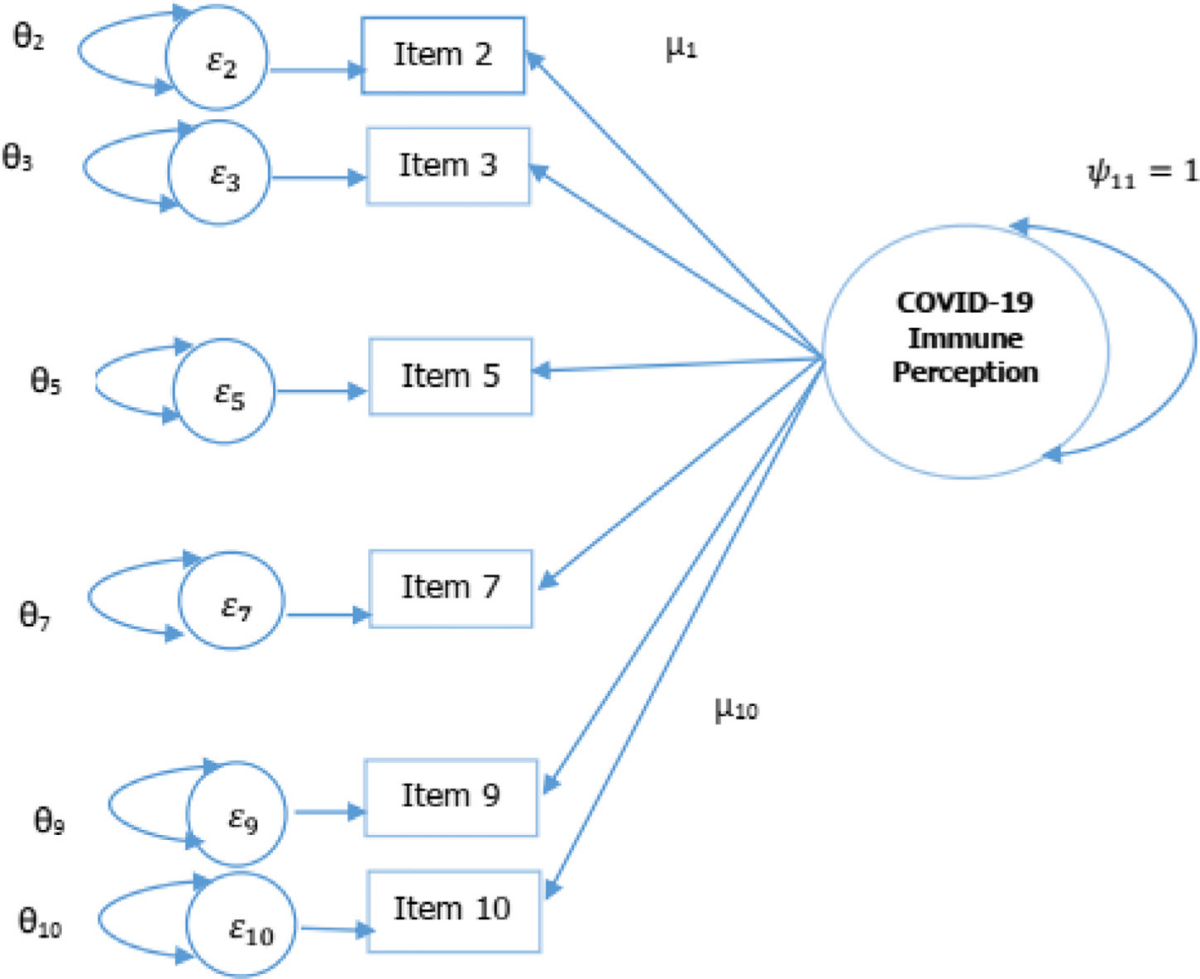
Path diagram *Key: Item2* – Africa Weather and humid system prevent the spread of COVID19, *Item3* – Africans are naturally resilient and resistant to most diseases, *Item5***–** Older people are most prone to the danger of COVID19, younger people are less prone, *Item7***–** Drinking hot water can prevent/resist/kill COVID19, *Item9***-** High-temperature cure COVID19, *Item10***-** China made the novel Coronavirus (COVID19) to become the global power.

Source: Authors

**Fig. 2:**
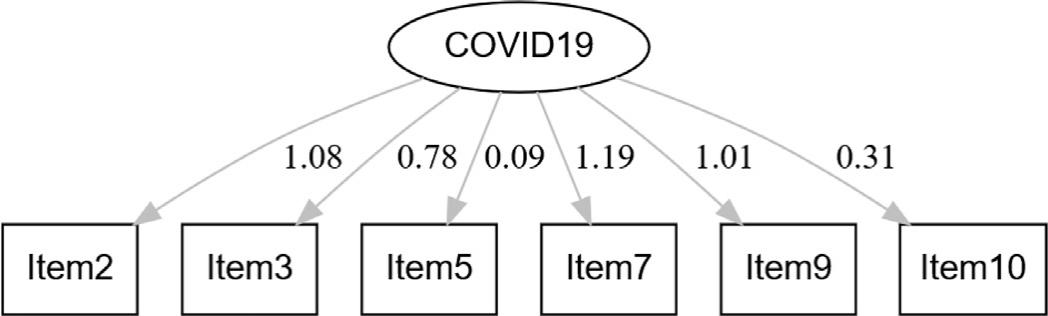
Path diagram output *Key: Item***2** – Africa Weather and humid system prevent the spread of COVID19, *Item3* – Africans are naturally resilient and resistant to most diseases, *Item5***–** Older people are most prone to the danger of COVID19, younger people are less prone, *Item7***–** Drinking hot water can prevent/resist/kill COVID19, *Item9***-** High-temperature cure COVID19, *Item10***-** China made the novel Coronavirus (COVID19) to become the global power.

*Source:* R output from Authors’ computation

Fig. 1 is the path diagram illustration of the six identified (most misleading) misinformation content with respect to the outcome in Table 2. The figure is used to depict the item path or contribution to the immune perception of COVID-19. As a tradition, Fig. 1 is necessary to arrive in Fig. 2. Hence, Fig. 2 presents the statistical result (in magnitude) of individual item contribution to promoting ignorance of COVID-19 pandemic in Africa.

Table 5 is a combination of all output or results of the confirmatory factor analysis conducted for the data. Table 5(a), presents confirmatory factor analysis (CFA) model fitness. Table 5(b) presents the individual contribution of the misinformation contents leading to immune perception against COVID-19 in Africa. Furthermore, Table 5(c) presents the value estimate of 1 against COVID-19 to prove that the level of perception of the virus remains constant or unchanged as long as this misinformation continues. In Table 5(d), the error correlation of the items is presented. Fig. 2 summarizes the output of the path diagram in Fig. 1.

**Table 5:** Items contribution.

(a): Model fitness						
Statistics	Value					
Comparative Fit Index (CFI)	0.92					
Tucker-Lewis Index (TLI)	0.90					
Root Mean Square Error of Approximation (RMSEA)	0.070					
90% CI – RMSEA *P*-value RMSEA <=0.05	[0.046, 0.099] 0.076					
Standardized Root Mean Square Residual (SRMR)	0.044					

(b): Latent variables						
Latent variables	Estimate(*μ_i_*)	Std.Er	z.val	P(>|z|)	Std.lv	Std.all
COVID-19						
Item2	1.083	0.092	11.740	0.00	1.083	0.604
Item3	0.777	0.095	8.140	0.00	0.777	0.421
Item5	0.092	0.073	1.258	0.209	0.092	0.067
Item7	1.187	0.094	12.640	0.00	1.187	0.657
Item9	1.011	0.089	11.373	0.00	1.011	0.583
Item10	0.310	0.090	3.457	0.001	0.310	0.183

(c): Variances						
Latent variables	Estimate(*μ_i_*)	Std.Er	z.val	P(>|z|)	Std.lv	Std.all
Item2	2.045	0.180	11.390	0.00	2.045	0.636
Item3	2.804	0.195	14.404	0.00	2.804	0.823
Item5	1.890	0.118	16.059	0.00	1.890	0.996
Item7	1.856	0.187	9.903	0.00	1.856	0.569
Item9	1.983	0.167	11.896	0.00	1.983	0.660
Item10	2.765	0.175	15.826	0.00	2.765	0.966
COVID-19	1.00				1.00	1.00

(d): Residual						
Latent variables	Item2	Item3	Item5	Item7	Item9	Item10
Item2	0.00					
Item3	0.291	0.00				
Item5	0.165	0.038	0.00			
Item7	−0.09	−0.108	−0.060	0.00		
Item9	−0.056	−0.233	−0.075	0.166	0.00	
Item10	−0.021	0.285	−0.192	−0.031	−0.075	0.00

*Key: Item2* – Africa Weather and humid system prevent the spread of COVID19, *Item3* – Africans are naturally resilient and resistant to most diseases, *Item5***–** Older people are most prone to the danger of COVID19, younger people are less prone, *Item7***–** Drinking hot water can prevent/resist/kill COVID19, *Item9***-** High-temperature cure COVID19, *Item10***-** China made the novel Coronavirus (COVID19) to become the global power.

Supplementary data and materials include the misinformation content responses before score titled “misinformation Raw data”; raw data from the survey, titled “perception of COVID-19 disease”; R script for the confirmatory factor analysis (CFA; **M 2**); and questionnaire for the survey in pdf form. All there are also available in the Mendeley repository.

## 2 Experimental design, materials, and methods

We developed ten items (misinformation contents) about COVID-19 which have formed major debates on African media space. These questions were diligently followed from the onset of the pandemic from all media platforms (between January 20 and March 25, 2020) as most circulated and conceived among Africans. We conducted opinion polls (oral engagements) on Kenyatta University (KU) Campus in Nairobi (between February 1 and February 25, 2020) as first case contact to access the general belief about the misinformation about COVID-19 pandemic spreading on social media space. KU like other African higher institutions has a large population of international students from all across Africa (making up to 90% of foreign students) and other foreign nationals including Europe, Asia, and America. Essentially, these questions were coined from the misinformation shared and conceived by a larger percentage of African social media users.

Apart from our initial poll on the KU campus, we also follow posts on social media space (Facebook, Whatsapp, Tweeter, YouTube, and E-news) between January 20 and March 30, 2020, and make records of several post followers, likes, retweets/shares, seen and read. By March 30, 2020, we made a compilation of the top ten misinformation contents about COVID-19 and sent out an e-questionnaire through Whatsapp and Email. We also shared the questionnaire with individuals on various international Whatsapp Groups and appeal with them to also share with their countrymen and women to gain wider coverage. The questionnaire is restricted to only persons of Africa descent. We also did a sample of all the misinformation on different fact check websites (see, e.g., https://www.hsph.harvard.edu/india-center/myths-vs-facts/) to see how relevant the questions are to the study.

## 3 Coverage & limitation

We are unable to cover a very large population (*n* = 563 responses), however, the sample space of the respondents covers all the regions of Africa and we received responses from 21 Africa countries. We received 66.7%, 12.7%, 4.6%, 15%, and 1% responses from West Africa, Central Africa region, Southern Africa, East Africa, and North Africa respectively. The majority of our respondents (93.4%) have a tertiary education (mostly postgraduate – 54.5%) compare to 5.9% high school leavers, 0.4% of primary school leavers, and 0.3% of persons without formal education. Similarly, the age distribution shows that 21.6%, 21%, 17.8%, 13.4%, 15.2%, 8.2%, 1.6%, and 0.7% are between the ages of 18 and 24 years, 25–29 years, 30 and 34 years, 35 and 39 years, 40 and 49 years, 50 and 59 years, 60 and 69 years, and 70 years and above respectively. Among these are 34.1% females and 65.9% males. The majority are Nigerians (56%), 14.1% are Cameroonians, 8.7% Ghanaians, 9.3% Kenyans, 2% South Africans, 2.1% DR Congolese, 1.6% Tanzanians, 1.2% Rwandans, 0.4% Burundians, and others are; Gambians, South Sudanese, Zimbabweans, Chadians, Zambians, Congolese, Botswana's, Sudanese, Comoros, Sierra Leoneans, Malawians, and Ugandans.

## 4 Methods

### 4.1 M 1: question module of the exploratory techniques

All responses were coded on a five-point Likert scale. Response options for all questions were “strongly agree”, “agree”, “neutral”, “disagree” and “strongly disagree”. The data aims to examine how misinformation contents promote people's poor perception of COVID-19 in Africa. Thus, strongly agree or agree implies "being influence by" and strongly disagree or disagree implies "Not being influence by" the misinformation contents. We assume being “neutral” as undecided and could go either way. We score each response on a scale of 4, 4, 1, 0, and 0 respectively. That is, the score of '4’ implies that the item promotes ignorance about the pandemic, score of ‘0′ implies the item does not promote ignorance about the pandemic, and the score of ‘1′ implies that the item neither. The range is computed by multiplying the score by the total number of items (*m* = 10). See Eq. (1) for the magnitude score formula. In essence, the aim is to score individual response per item and measure the magnitude of influence of the item on ignorance about the pandemic on the whole sample. Hence, each participant is scored as in Table 1.

Similarly, for Table 2, the range is between “0 and 40″. Score “below 10″ is considered as misinformation content “Not promoting” ignorance about COVID-19 and “above 10″ is considered as misinformation content “Promoting” ignorance about COVID-19.

Thus, the magnitude score (*MgS*) is computed as follows:

(1) $$MgS=\;{\left(\left(\frac{\sum_{i=1}^{n}R_{ij}}{n}\right)x\;I_{m}\right)}_{j\in m}$$

where *n* – is the total number of respondents, *R_i_* – is the *j*th item score of the *i*th respondent, *m* – number of items, and *I_m_*- is the total number of items; ∀*i* counts against *j*th item at a time. This means that we compute (as in 1) for the *i*th score of the respondents per *j*th item.

Test of the significant (presented in Table 3) influence of the misinformation on the immune perception against COVID-19 in Africa, we conduct a Chi-square test statistics on each of the items. The reference point is the *P*-value. The Chi-square value is just mathematical output which is used in obtaining the *P*-value by the computer. Thus, it has nothing to do with the magnitude score in Table 2. Therefore, if *P*-value is less than α (= 5%), the null hypothesis (H_0_) is rejected. That means that the misinformation content/item promotes ignorance about the pandemic. See Table 3 for the *P*-value of each item.

Hypothesis:

H_0_: Misinformation content/items do not promote ignorance about the pandemic;
H_1_: Misinformation content/items promote ignorance about the pandemic;
α = 5%.

Lastly for this section, we conduct a correlation analysis to examine the joint influence of items (misinformation contents) on immune perception against the novel COVID-19. This is presented in Table 4.

### 4.2 M 2: analytical techniques

For the second phase of the data analysis, a more advanced technique is considered to assess the effects of the misinformation items on the COVID-19 immune perception. Hence, we adopt the confirmatory factor analysis (CFA) techniques. CFA is a common structural equation model technique in which one specifies how observed variables (misinformation contents) relate to assumed latent (COVID-19 immune perception) variable(s) [4,5]. That is, it allows us to evaluate the structure or extent of the latent variable base on all available evidence. Following the classification in the exploratory section (see Table 2), we have the “six items one factor” CFA model. Although all the items significantly promote ignorance of the pandemic, the choice of the six items is based on the misinformation magnitude scale (in Table 2) and correlation result (in Table 4). This helps to avoid violation of some classical/statistical assumptions (such as multicollinearity etc.).

We adopt the variance standardization method. This method fixes the variance of each factor (items) to 1 but freely estimates all loadings and it is more robust for CFA of items greater than 3. In matrix notation, our CFA model is:

$$\sum \left(\theta \right)=\psi_{11}\;\left(\begin{array}{c} \mu_{1}\\ \mu_{2}\\ .\\ .\\ .\\ \mu_{6} \end{array}\right)\;\left(\mu_{1},\;\mu_{2},\ldots ,\;\mu_{6}\right)\;\left(\begin{array}{c} \theta_{11},\;0,\;\ldots 0\\ 0,\;\theta_{22},\;\ldots 0\\ 0,\;\theta_{33},\;\ldots .\;0\\ \ddots \\ 0,\;0,\;\ldots .\theta_{66} \end{array}\right)$$

Mathematically,

(2) $$\sum \left(\theta \right)=\;\Delta \Psi \Delta^{I}+\;\Theta_{\in }$$

Where; ∑(*θ*) – is the observed covariance matrix, Δ – is the factor loading matrix, Ψ – is the variance-covariance matrix of the latent factors, and Θ_ ∈ _ - is the variance-covariance matrix of the residuals.

### 4.3 M 2.1: path diagram

The path diagram gives a visual representation of our CFA model because it is a symbolic one-to-one visualization of the measurement model and the model-implied covariance. For general understanding, the circle represents the latent variable – COVID-19 immune perception, squares represent observed items, the one-way arrow represents paths and two-way arrows represent either variances or covariances. Following our matrix notation above, the CFA path diagram is presented below and we adopt R programming for the CFA analysis.

Table 4 presents the summary output of the confirmatory factor analysis. Each section within the table has a highlight that gives a basic description of their content as explained under data description.

## Declaration of Competing Interest

The authors declare that they have no known competing financial interests or personal relationships which have, or could be perceived to have, influenced the work reported in this article.
